# Distribution and potential risk factors of bisphenol a in serum and urine among Chinese from 2004 to 2019

**DOI:** 10.3389/fpubh.2024.1196248

**Published:** 2024-01-31

**Authors:** Wenjing Zhang, Yanting Li, Tao Wang, Xinglin Zhang, Jianzhong Zhang, Xiaoya Ji, Lin Lu

**Affiliations:** Department of Occupational and Environmental Health, School of Public Health, Qingdao University, Qingdao, China

**Keywords:** urine bisphenol A, serum bisphenol A, Chinese population health, Monte Carlo simulation, risk factors, environmental pollutants, endocrine disruptor

## Abstract

**Background:**

Bisphenol A (BPA) is an oil-derived, large-market volume chemical with endocrine disrupting properties and reproductive toxicity. Moreover, BPA is frequently used in food contact materials, has been extensively researched recently, and widespread exposure in the general population has been reported worldwide. However, national information on BPA levels in general Chinese people is lacking.

**Methods:**

This study collected and analyzed 145 (104 in urine and 41 in serum) research articles published between 2004 and 2021 to reflect the BPA internal exposure levels in Chinese populations. The Monte Carlo simulation method is employed to analyze and estimate the data in order to rectify the deviation caused by a skewed distribution.

**Results:**

Data on BPA concentrations in urine and serum were collected from 2006 to 2019 and 2004 to 2019, respectively. Urinary BPA concentrations did not vary significantly until 2017, with the highest concentration occurring from 2018 to 2019 (2.90 ng/mL). The serum BPA concentration decreased to the nadir of 1.07 ng/mL in 2011 and gradually increased to 2.54 ng/mL. Nationally, 18 provinces were studied, with Guangdong (3.50 ng/mL), Zhejiang (2.57 ng/mL), and Fujian (2.15 ng/mL) having the highest urine BPA levels. Serum BPA was investigated in 15 provinces; Jiangsu (9.14 ng/mL) and Shandong (5.80 ng/mL) were relatively high. The results also indicated that males’ urine and serum BPA levels were higher than females, while the BPA levels in children were also higher than in adults (*p* < 0.001). Furthermore, the volume of garbage disposal (*r* = 0.39, *p* < 0.05), household sewage (*r* = 0.34, *p* < 0.05), and waste incineration content (*r* = 0.35, *p* < 0.05) exhibited a strong positive connection with urine BPA levels in Chinese individuals.

**Conclusion:**

Despite using a data consolidation approach, our study found that the Chinese population was exposed to significant amounts of BPA, and males having a higher level than females. Besides, the levels of BPA exposure are influenced by the volume of garbage disposal, household sewage, and waste incineration content.

## 1 Introduction

Bisphenol A (BPA) is an artificial chemical compound with high production volume, predominantly used in manufacturing polycarbonate (PC) plastics, polysulfones, and epoxy resins (1, 2). Several studies suggest that BPA is widely applied in producing varying consumer products, such as thermal paper, can coatings (3, 4), and BPA can leach from food and beverage containers, dental sealants, and other composites (5). Due to its widespread application, BPA is detected in water (freshwater, seawater, sewage, drinking water), soil or sediment, atmosphere, food, garbage (4, 6), and in humans. Furthermore, since 2007, Asia has become a significant BPA production and consumption region. At the end of 2018, BPA consumption was 1.43 million tons in China, making it the largest producer of BPA globally (7).

Humans are exposed to BPA through various pathways, including food or drinking water (8), skin contact, and breathing (9), in which dietary intake is the main pathway. Exposure to airborne BPA cannot be ignored, as shown by Ribeiro et al. (10). The study conducted by Rudel et al. revealed the presence of BPA in 86% of house dust samples, with concentrations ranging from 0.2 to 17.6 μg/g (11). In the urban outdoor environment, this compound has been detected in air samples at an average level of 0.51 ng/m^3^ with mild seasonal variations observed (12). According to a Chinese study on the causes of urinary BPA exposure in young adults, dietary consumption, indoor dust, paper goods, and personal care items contributed 72.5, 0.74, 0.98, and 0.22% of the overall exposure dose, respectively (13). Several epidemiological research have suggested that fatty foods, bracelets, and socks may be sources of BPA exposure for children (14–16). Moreover, in a human study embedded as part of the Europe project EuroMix (“European Test and Risk Assessment Techniques for Mixtures”) it was determined that diet and thermal paper (TP) were the factors most responsible for BPA exposure (17). Occupational exposure, in addition, is an important mode of exposure to BPA. The study conducted by He et al. showed that workers in epoxy resin and BPA manufacturing factories are occupationally exposed to BPA at high levels (18).

Upon oral treatment, pharmacokinetic investigations have shown that BPA is swiftly and effectively absorbed in the gastrointestinal tract. It is first metabolized by the gut wall and liver, where its primary metabolite BPA-glucuronide is produced. BPA-glucuronide is rapidly filtered from the blood by the kidneys and excreted in urine (19). Consequently, BPA has been detected in body fluids, including saliva, urine, serum, plasma, placental tissue, umbilical cord serum, placenta and breast milk (20, 21). Though the half-life of BPA in human urine and blood is 6 h and 5.3 h, respectively (22, 23), urine and serum are widely used biological samples for biomonitoring (19).

Due to its endocrine disrupting properties and reproductive toxicity, BPA shows substantial damage to tissues and organs of the body, including those of the cardiovascular, reproductive, immune (24, 25), respiratory, digestive, and neuroendocrine systems (26–30). Previous epidemiological and toxicological studies demonstrated that exposure to BPA can cause endocrine disruption in humans (31), development of obesity (32), diabetes (33), cardiovascular disease (34, 35), etc. Sandra studies indicate that there were no significant differences in BPA exposure levels among the general population based on sex, geographic region, or analysis technique (36). For the moment, developed countries such as the United States and Canada have carried out long-term and systematic biomonitoring programs for urine BPA in their populations. China has carried out biological monitoring of BPA exposure levels in populations in localized areas, such as Shanghai (37, 38), Jiangsu (39), and Guangdong (39, 40) provinces, so there is a lack of exposure level data for the entire Chinese population.

There is currently no long-term detection and spatial variation trend of BPA exposure in the Chinese population. The study used Monte Carlo simulation to estimate urine and serum BPA levels in the Chinese population, which our group had previously established and validated. We used Monte Carlo simulation to examine BPA’s regional and temporal distribution in urine and serum from 2004 to 2019. Additionally, relevant potential risk factors influencing urine and serum BPA levels were investigated.

## 2 Methods

### 2.1 Aim

The purpose of this study was to analyze the level of BPA exposure in the Chinese population from 2004 to 2019. And analyze the relationship between demographic factors such as age, gender, and region and the level of BPA exposure. In addition, the relationship between BPA exposure levels in the human body and BPA exposure in the environment were evaluated.

### 2.2 Design

We searched for literature on BPA exposure levels in the Chinese population from May 2011 onwards. Monte Carlo simulation was used to integrate and analyze the data.

### 2.3 Sample

PubMed, China National Knowledge Infrastructure (CNKI), Weipu (VIP), and Wanfang Data was selected as the academic publication source in this study.

### 2.4 Literature search

Four databases, such as PubMed, CNKI, VIP, and Wanfang Data, were searched from inception to May 1, 2021. We identified the following keywords: (a) “bisphenol A” and “urine” and “human” (b) “bisphenol A” and “serum” and “human” was decided as the keyword to search the Chinese database. Literature in English searches for anthropological studies using “bisphenol A” and “human” and “China” or “bisphenol A” and “Chinese” as keywords. The duplicate articles were excluded from the four databases.

Inclusion criteria were as follows: (1) All the subjects were Chinese population; (2) The study population were not patients with certain BPA-related diseases, such as obesity, asthma, thyroid disorders, neurobehavioral disturbances, changes in reproductive function, abnormal mammary gland development, and cognitive dysfunctions; (3) Subjects were not with high BPA exposure history (described as living or working in areas of high BPA concentration); (4) The test samples were urine and serum, and strict quality control was used in the detection procedure. Data were filtered using the following exclusion criteria: (1) Research performing animal tests; (2) Studies including case reports, conference or poster abstracts, reviews, letters, or articles without containing original data; (3) Studies on substitutes for BPA.

**Figure 1:**
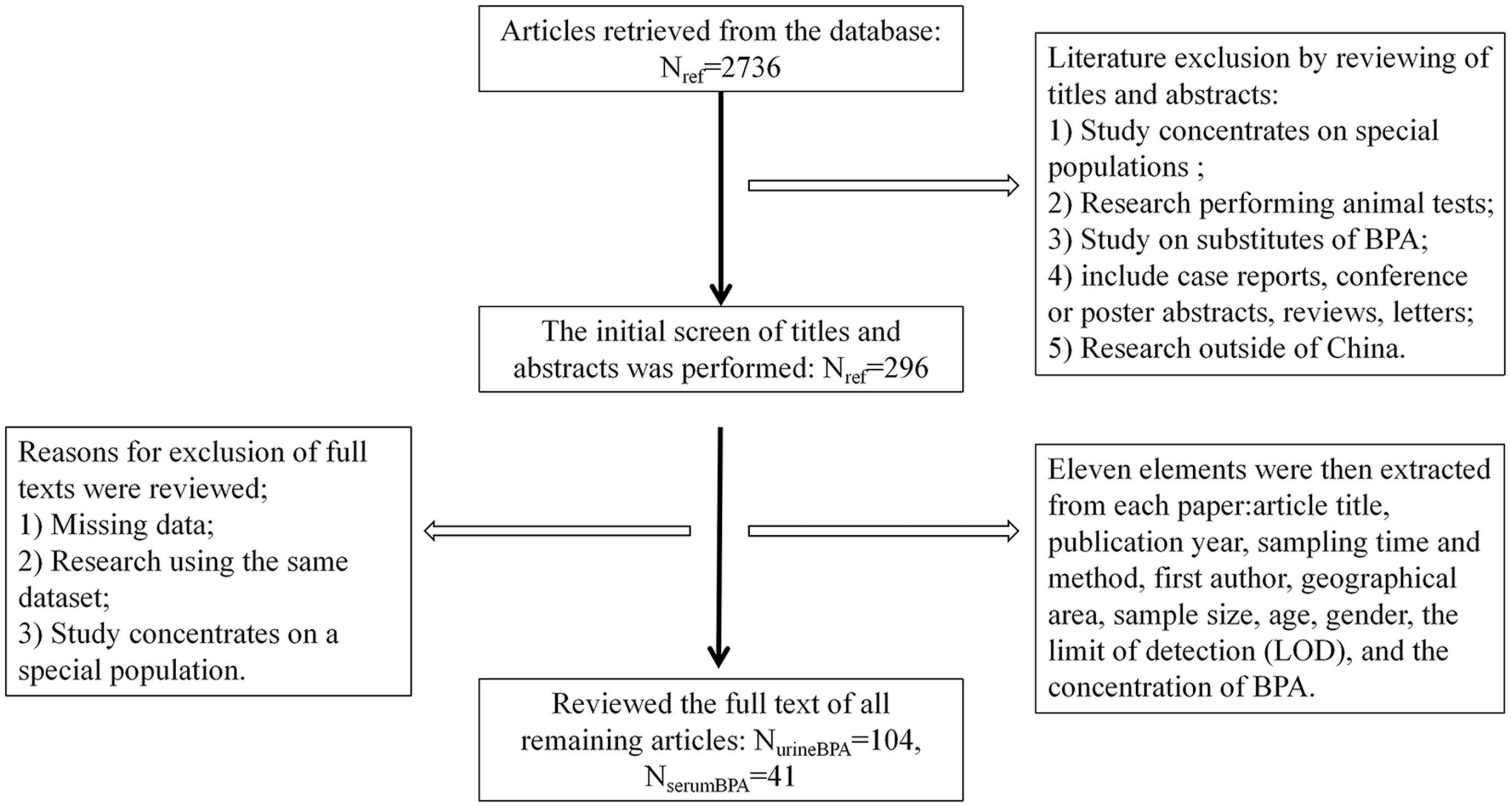
Steps taken to identify articles that met our inclusion–exclusion criteria. Urine andserum BPA data were collected from Weipu (VIP) databases, PubMed, China National Knowledge Infrastructure (CNKI), and Wanfang. One hundred forty-five papers reported the urine and serum BPA levels from 2004 to 2019 (sampling time).

A total of 145 articles published from 2004 to 2021 (including 104 on urine BPA and 41 on serum BPA) were collected, including 78 pieces of Chinese literature (50 on urine BPA and 28 on serum BPA) and 67 parts of English literature (54 on urine BPA and 13 on serum BPA) (see Figure 1). The sampling time ranged from 2004 to 2019. These articles included 64,893 subjects with sample sizes ranging from 10 to 3,426 each (see Supplementary Tables S1, S2).

### 2.5 Data extraction

Eleven elements were then extracted from each paper and entered into an Excel spreadsheet: article title, publication year, sampling time and method, first author, geographical area, sample size, age, gender, the limit of detection (LOD), and the BPA concentration (see Figure 1). We divided the available urine BPA and serum BPA data into five periods (2006–2008, 2009–2011, 2012–2014,2015–2017, 2018–2019) based on sampling time.

The National Health and Nutrition Examination Survey (NHANES) 2003–2016 provided the American urine BPA data (41). The Fifth Report on Human Biomonitoring of Environmental Chemicals in Canada 2007–2017 is the source of Canadian urine BPA (42). The required data on garbage disposal, domestic sewage, and waste incineration were found in the China Statistical Yearbooks (2006–2019) (43).

### 2.6 Data processing

#### 2.6.1 Unit conversion of urine and serum BPA level

In this study, uniform unit was ng/mL. Other units were converted to ng/mL, e.g., 1 μg/L = 1 ng/mL; 1 ng/mL = 100 ng/dL; 1 ng/mL = 1,000 ng/L.

#### 2.6.2 The calculation of arithmetic means

For most studies, the median and interrogative range values were presented, and to a lesser extent, geometric mean or arithmetic means. The appropriate Monte Carlo simulation formula was determined by consideration for whether the data fit a normal distribution or log*-*normal distribution or not. We convert the median to arithmetic mean (https://smcgrath.shinyapps.io/estmeansd/).

### 2.7 Calculation of standard deviation

The standard deviation of urine and serum BPA level not available in articles was estimated by Equation (44) as follows (45):


$$SD=\sqrt{N}\left(U_{CI}-L_{CI}\right)÷3.92$$

Where *N* is the sample size; *U_CI_* and *L_CI_* are the upper and lower 95% confidence intervals, respectively.

### 2.8 Monte Carlo simulation

We used excel software to accomplish Monte Carlo simulation analysis on the data. The workflow of our approach was composed of three steps: (1) composing a mathematical model for probability simulation; (2) abstracting the simulated random numbers; and (3) arranging statistics and getting the solution to the problem (44).

For the original data of normal distribution and lognormal distribution, we adopted different calculation methods. Formulae used were as follows:


=MAX(0.5,NORM.INV(RAND(),AM,SD))

=MAX(0.5,LOGNORM.INV(RAND(),Ln(GM),Ln(GSD)))

Where NORM.INV and LOGNORM.INV denotes interval points that return a given probability normal distribution or lognormal distribution, respectively. RAND returns evenly distributed random numbers greater than or equal to 0 and less than 1. The MAX function ensured that the urine and serum BPA levels were greater than 1/2 LOD. Values were calculated in Excel (Microsoft). Simulations were computed by 100 times to ensure their exactitude (see Supplementary Figure S1).

### 2.9 Statistical analyses

Statistical analysis was conducted using SPSS version 26 (IBM. Armonk, NY, United States). Figures were prepared using GraphPad Prism 8.3.0 and ArcGIS 10.6. The Mann–Whitney U test was utilized to compare differences between two independent groups. Correlations were evaluated by Pearson’s correlation coefficient (r). *p*-values < 0.05 were considered statistically significant. Urine and serum BPA results were transformed using natural logarithms for data analysis.

## 3 Results

### 3.1 Trends of urine BPA and serum BPA concentration in general Chinese population from 2004 to 2019

**Figure 2:**
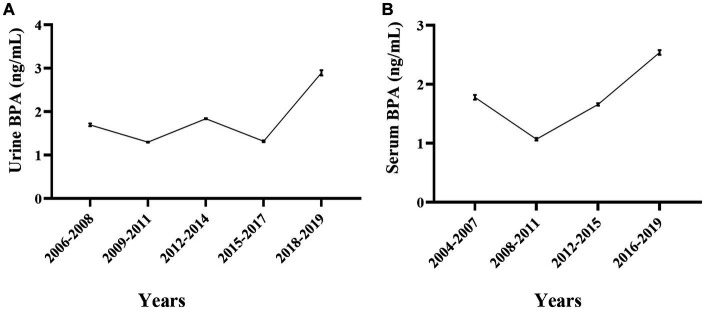
The temporal trend of urine ad serum BPA levels in general Chinese population from 2004 to 2019. **(A)** Overview of the general Chinese population’s urine BPA levels in different periods. **(B)** Overview of the general Chinese population’s serum BPA levels in different periods.

The Chinese population’s urine and serum BPA concentration was calculated from the new database, which was generated by Monte Carlo simulation. Figure 2 depicts the urine and serum BPA concentrations in different time period (2006–2008, 2009–2011, 2012–2014, 2015–2017, 2018–2019 for urine BPA and 2004–2007, 2008–2011, 2012–2015, 2016–2019 for serum BPA). The geometric mean of serum BPA concentration decreased from 1.78 ng/mL in 2004–2007 to 1.07 ng/mL in 2008–2011, while the geometric mean of serum BPA concentration increased from 1.66 ng/mL in 2012–2015 to 2.54 ng/mL in 2016–2019 (see Supplementary Table S3).

### 3.2 Geographical variation of urine and serum BPA concentration

**Figure 3:**
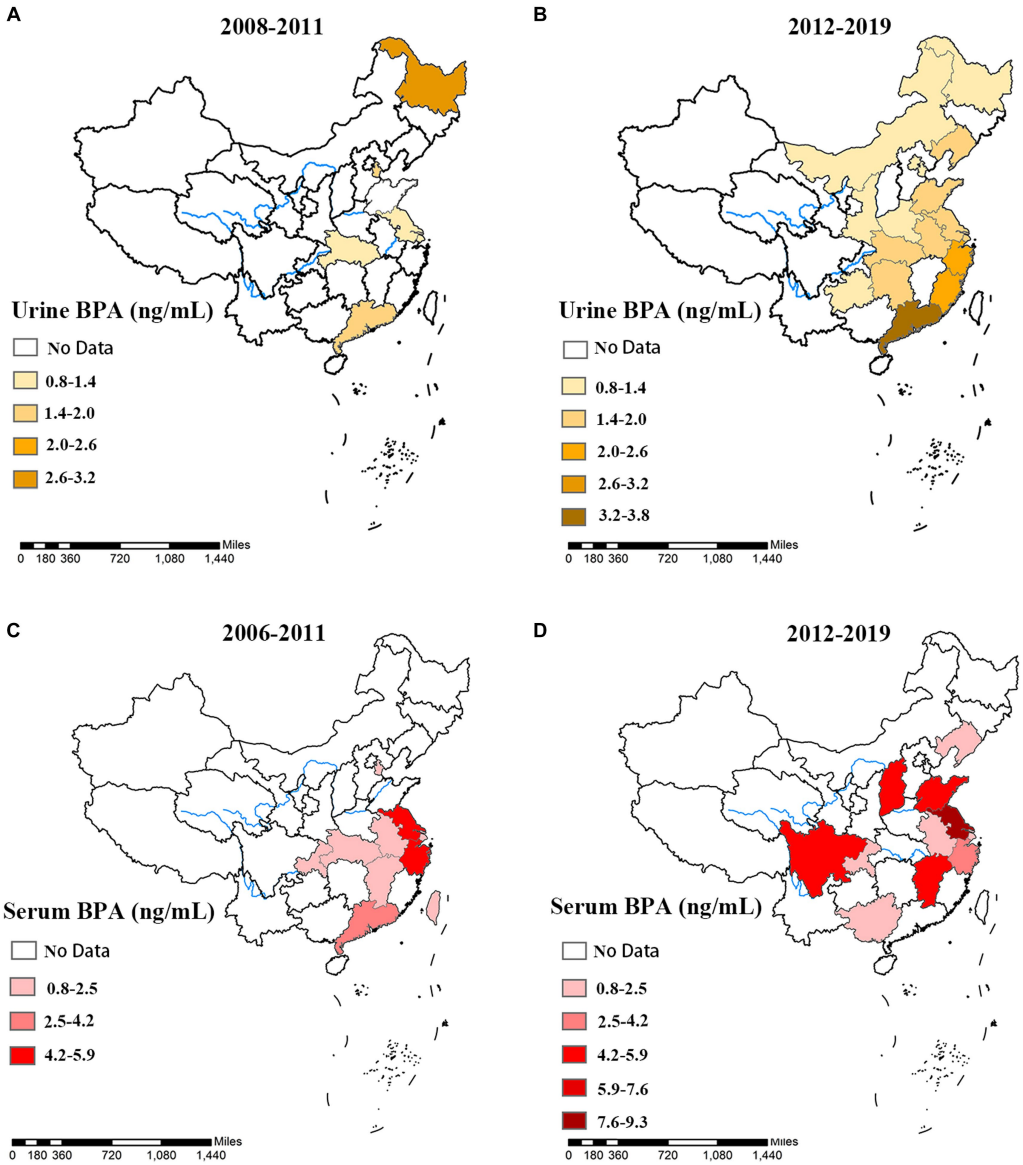
The spatial and temporal distribution of Urine and serum BPA concentration of different provinces in China. Urine BPA concentration in different provinces, **(A)** 2008–2011, and **(B)** 2012–2019 were exhibited. Serum BPA concentration in different provinces, **(C)** 2006–2011, and **(D)** 2012–2019 were exhibited.

Figure 3 depicts the urine and serum BPA concentrations in different provinces across two time periods (2008–2011, 2006–2019 for urine BPA and 2006–2011, 2012–2019 for serum BPA). Urine BPA concentrations (2008–2011) and serum BPA concentrations (2006–2011) were detected mostly in coastal locations (see Figures 3A,C), with the area expanding inland from 2012 to 2019 (see Figures 3B,D). Guangdong (3.5 ng/mL), Zhejiang (2.57 ng/mL), and Fujian (2.15 ng/mL) had greater urine BPA levels than the other provinces, whereas Inner Mongolia (0.8 ng/mL) had a comparatively low level (see Supplementary Table S4). The levels of BPA in serum differed substantially between provinces. Jiangxi had the lowest serum BPA content (0.8 ng/mL), and Jiangsu had the highest (9.2 ng/mL) (see Supplementary Table S5).

### 3.3 Gender difference

**Figure 4:**
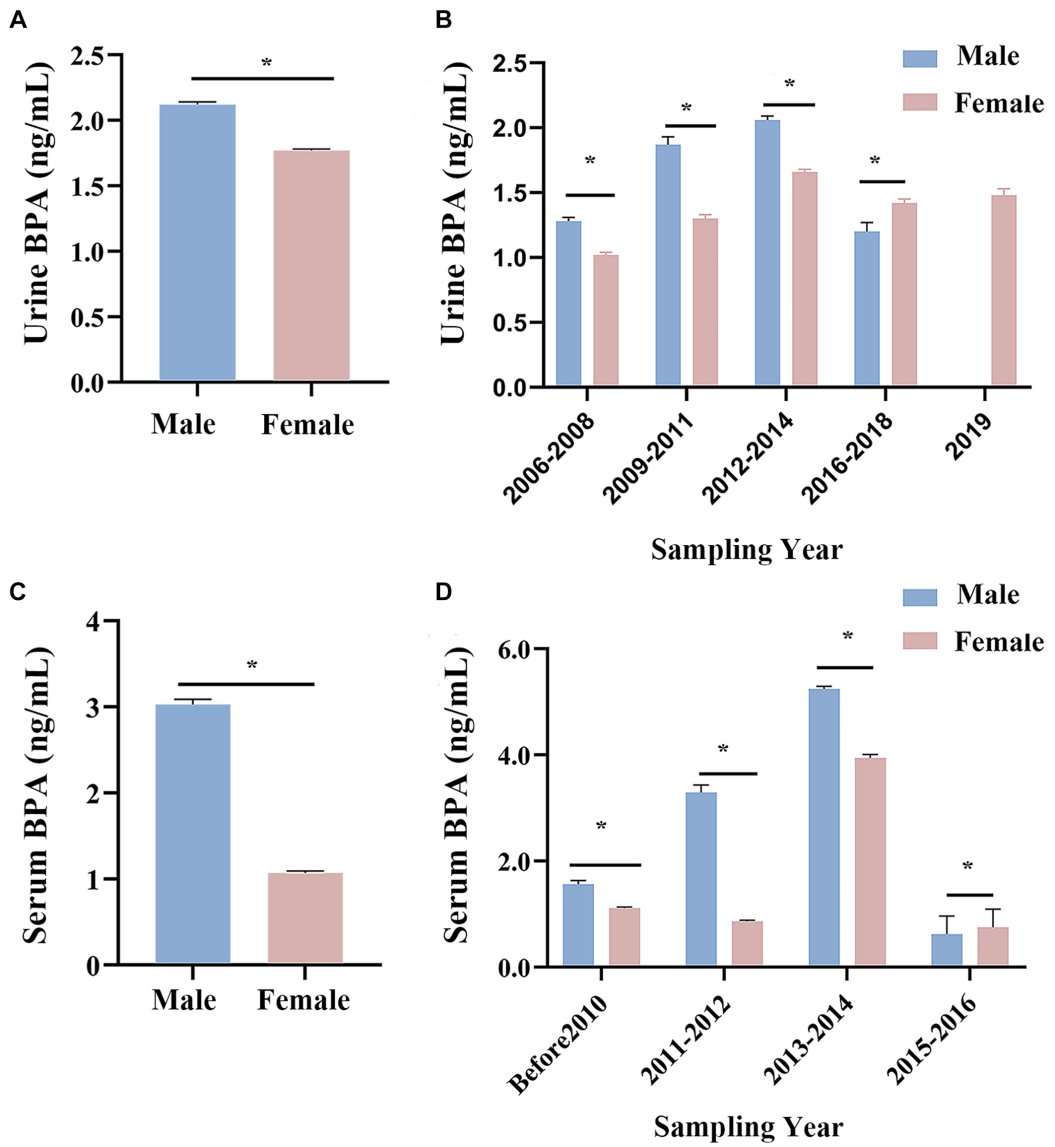
Changes in Chinese urine and serum BPA concentration by gender. **(A)** An overview of Chinese urine BPA concentration in different gender. **(B)** Histogram of Chinese urine BPA concentration of male and female in different time. **(C)** An overview of Chinese serum BPA concentration in different gender. **(D)** Histogram of Chinese serum BPA concentration of male and female in different time. *p < 0.05.

Males had higher urine and serum BPA concentrations than females (see Figure 4). Urine BPA concentrations in males and females in China were 2.12 ng/mL and 1.77 ng/mL, respectively, while serum BPA values were 3.03 ng/mL and 1.07 ng/mL (see Figures 4A,C, Supplementary Table S6). The Mann–Whitney U test showed that the BPA concentration of males was significantly higher than that of females (all *p* < 0.05). Additionally, slowly increase in urine and serum BPA concentration in both males and females was noted before 2014, with decrease gradually from 2015 to 2019 (see Figures 4B,D).

### 3.4 Age difference

**Figure 5:**
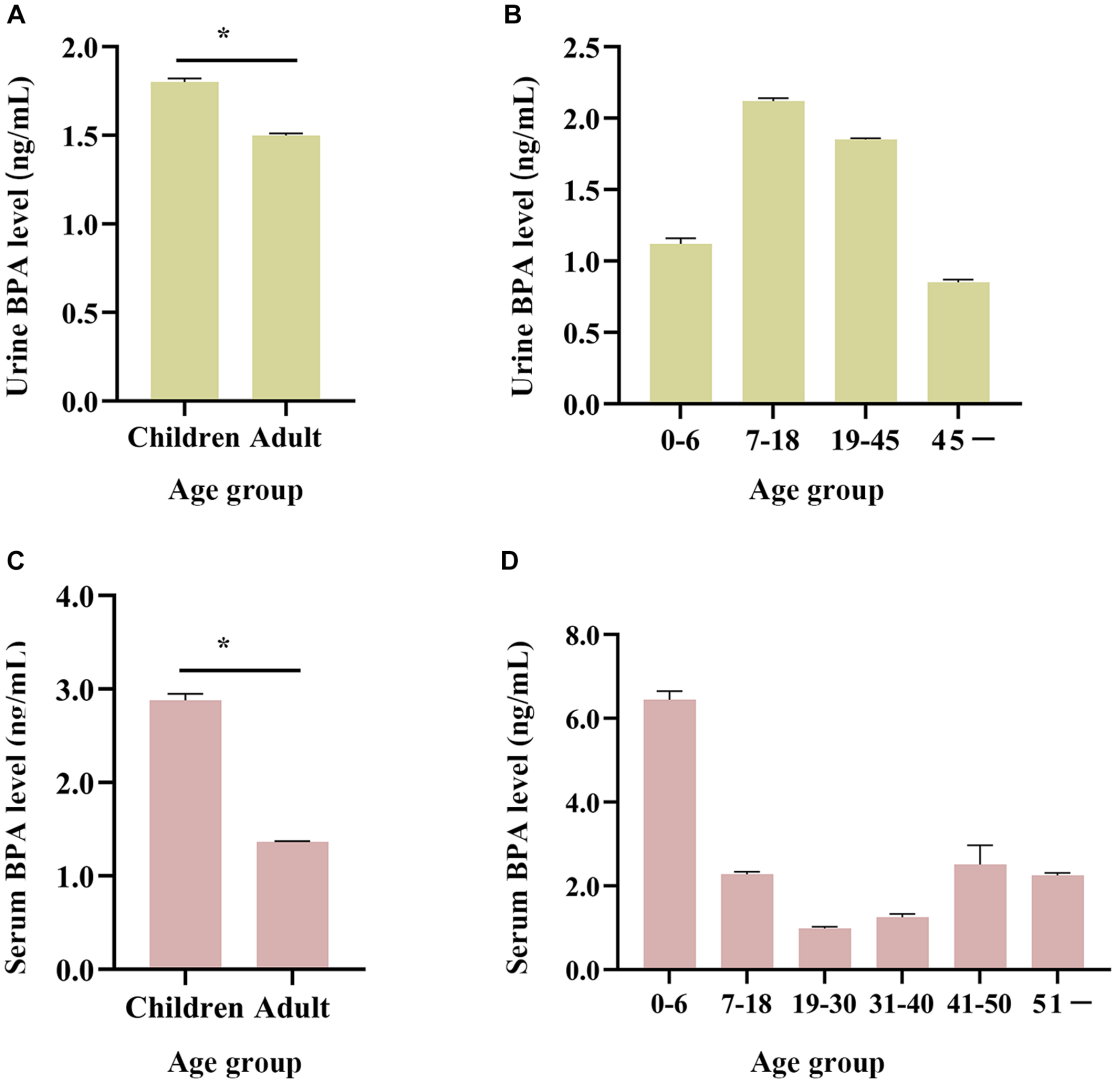
Age distribution of BPA levels in the general Chinese population (from 2004 to 2019). **(A,B)** Age changing trend of urine BPA concentration. **(C,D)** Age changing trend of serum BPA concentration. Children are 0–18 years old and adults are ≥19 years old. *p < 0.05.

Taking ages into consideration, urine and serum BPA concentrations in China among different age ranges were 1.80 ng/mL and 2.88 ng/mL in the group of 0–18 years, 1.50 ng/mL and 1.36 ng/mL in the group of 19 and above years old (see Figures 5A,C). The Mann–Whitney U test showed that the BPA concentration of children was significantly higher than that of adults (all *p* < 0.05). Besides, urine BPA concentration was higher in school-age and young adults compared with people of other ages, with the geometric mean of 2.12 ng/mL and 1.85 ng/mL, respectively (see Figure 5B, Supplementary Table S7). Serum BPA concentration was highest in groups of 0–6 years old, with a geometric mean of 6.44 ng/mL (see Figure 5D, Supplementary Table S7).

### 3.5 Comparison with U.S. and Canada

**Figure 6:**
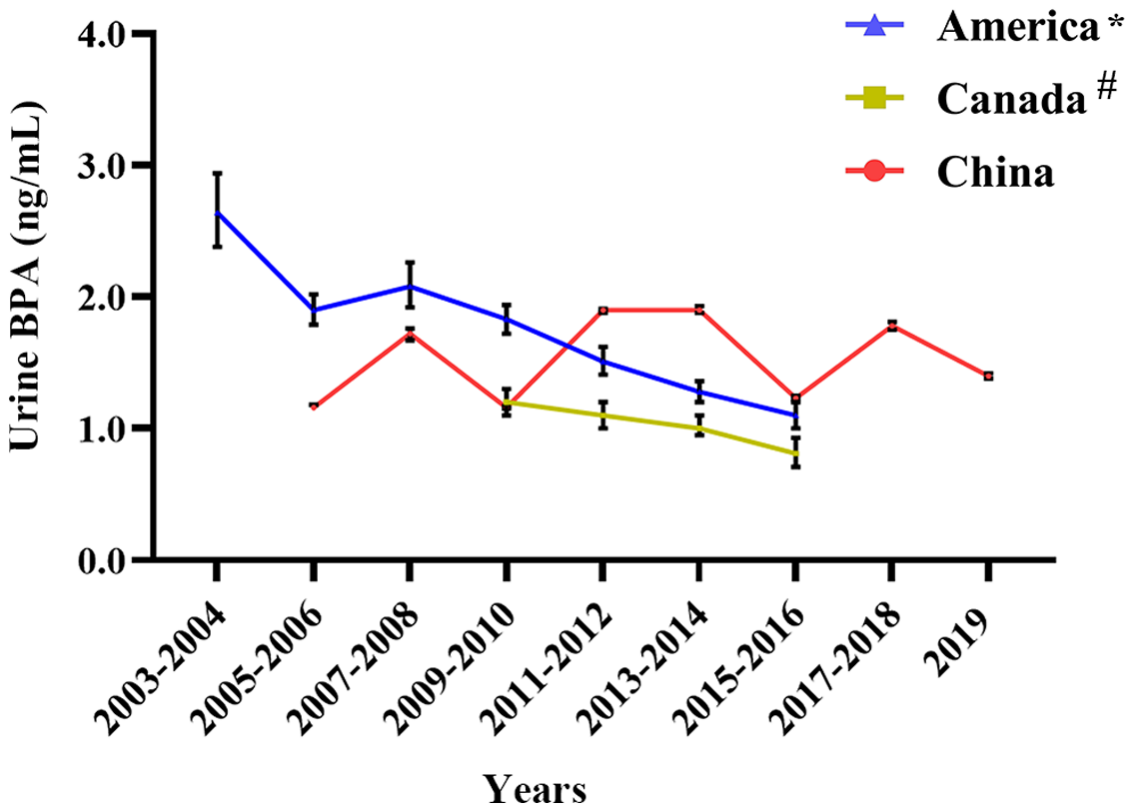
Urine BPA concentration in China compared with foreign countries. * Denotes that the source of American urine BPA data is NHANES 2003–2016; # Means that the data of urine BPA of Canadian are from The Fifth Report on Human Biomonitoring of Environmental Chemicals in Canada 2009–2016.

In the United States and Canada, urine BPA levels showed an apparent downward trend, while urine BPA concentrations of the Chinese exhibited a significant fluctuation (see Figure 6). Before 2011, the concentration of BPA in urine in China was lower than that in the United States. However, from 2011 to 2019, the concentration of urine BPA in China was higher than that in the United States and Canada (see Figure 6).

### 3.6 The association between urine BPA concentration in Chinese and the external environment

**Figure 7:**
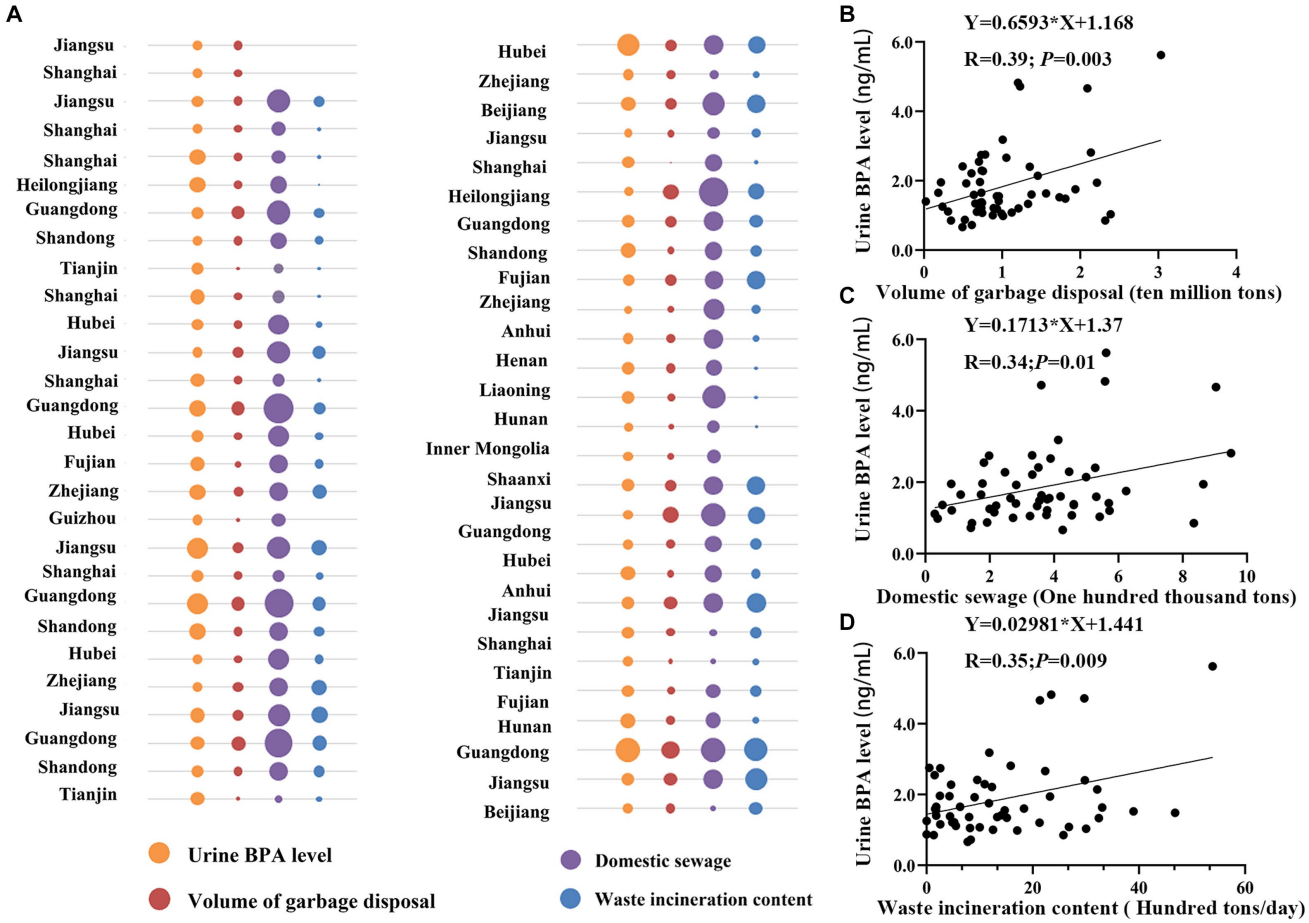
The correlation between urine BPA levels and potential risk factors. **(A)** Bubble chart; **(B)** volume of garbage disposal; **(C)** domestic sewage; **(D)** waste incineration content.

Environmental factors can influence humans BPA inhalation, ingestion, and skin absorption. As shown in Figure 7A, possible risk factors such as garbage disposal, residential sewage, and waste incineration content were connected to urine BPA concentration in Chinese provinces (see Supplementary Table S8). Guangdong had the highest urine BPA level, as well as the biggest volume of rubbish disposal and a high level of residential sewage and waste incineration. Domestic sewage levels are also higher in Heilongjiang and Zhejiang provinces. Furthermore, urine BPA levels revealed a strong positive connection with garbage disposal volume (*r* = 0.39, *p* = 0.003) (see Figure 7B), as did household sewage and waste incineration content (*r* = 0.34, *p* = 0.01; *r* = 0.35, *p* = 0.009) (see Figures 7C,D).

## 4 Discussion

Though BPA-related health problems have been attracting increasing attention (46), large-scale population studies with BPA exposure levels are still lacking in China. Several studies have shown that the average Chinese person had a high BPA exposure level (47). Furthermore, BPA has been widely investigated for its toxicity and shown adverse effects even at low-dose in animals and humans (48), making it a public health problem in China.

Urine and serum BPA level have acted as validated biomarkers to assess BPA exposure in humans. It mainly reflects short-term BPA exposure because of BPA’s relatively short-life in urine and serum. He et al. reported BPA concentration in urine and serum of 952 ordinary residents (49). In September 2010, Gao’s team conducted a national urine BPA level survey (13). However, most studies have been cross*-*sectional, and few have evaluated changes in human urine and serum BPA level over time. Based on the nationwide data investigation, our results suggested that the Chinese population’s urine BPA had a smooth fluctuation then increased to a high level, while serum BPA gradually increased after reaching its lowest concentration in 2011. Like the United States and Canada (50), China has begun to restrict the use of BPA. However, BPA has been banned in the use of baby bottles, but BPA was still permitted to be used in the production of food packaging materials, containers, and coatings. Before 2011, BPA was in considerable demand, but the imports of BPA were on the decline (51). It has been shown that BPA imports were 8.8% lower in 2008 than in 2007 (52). Additionally, demand for BPA is growing in industries such as home appliances, electronics, urban construction, and automobiles (53).

Instead of focusing on the whole population in China, these present studies paid more attention to localized areas, such as Shanghai, Jiangsu, and Guangdong. This study collected urine and serum BPA data from different provinces. Through comparative analysis, we found that the Chinese population’s BPA concentration of biological samples was predominantly distributed in the coastal areas (2004–2011). However, since 2012, the monitoring of BPA has expanded to inland areas. An important reason is that the abundance of seafood products may contain BPA in coastal areas. On the one hand, BPA manufacturers are mainly distributed in Jiangsu, Shanghai, and Zhejiang, among which a chemical company in Jiangsu is the one of the major thing BPA manufacturer in China (54). On the other hand, BPA is extensively used in the production of plastics and microplastics detected in 80–100% of seafood in southeastern China, leading to high levels of BPA in marine fish (55, 56). Additionally, in the atmosphere, BPA concentration is still at a high level in southeast China (57).

In general, the difference in gender and age has influenced the distribution of BPA. This study *shows that* urine and serum BPA concentrations were higher in males than females, *which* was in agreement with the previous studies (49). The elevated level of BPA in male urine may be associated with the glucuronide-conjugated coupling of BPA in urine (58). Moreover, a study in Korea found that gender differences in serum BPA concentrations may involve androgen-related BPA metabolism (59). In the 2015–16 period serum levels were higher in women and in the 2016–18 period females exhibited higher urine levels compared to other periods. On the one hand, the sample size in the urine BPA study conducted between 2016 and 2018 on women (*n* = 2,204) was significantly larger compared to men (*n* = 439). The size of the serum Bisphenol A exposure sample was smaller in 2015–2016. On the other hand, factors that cannot be assessed, such as a person’s employment, lifestyle choices, or even biological factors, were significant in this regard. BPA levels were two times higher among female employees, according to research by González et al. (0.68 g/L in men and 1.20 g/L in women), although this variation did not achieve statistical significance (*p* > 0.05), which may be connected to the fact that the study’s gender-specific workplaces were different (60).

Otherwise, we also found that urine and serum BPA concentrations were higher in children than that in adults. High concentrations of BPA in children may be associated with their high food consumption (such as fried food and snacks), relevant product usage (such as plastic toys), air inhalation rates in relation to their body weight and different toxic dynamic of their absorption, distribution, metabolism of BPA (61–63). Sugar and confectionery consumption were positively correlated with serum BPA levels in a Spanish sample (64). Additionally, Larsson et al. reported greater BPA levels in kids who consumed chocolate frequently and hypothesized that this could be due to a higher frequency of food consumption tainted by food packaging materials (65). There is BPA levels discrepancy between urine and serum values in the age groups 0–6, and 7–18 years. Children under the age of six are more probable to be exposed to plastic toys, and school-age children between the ages of seven and 18 spend more time in school as a result of dietary and airborne BPA exposure. These differences in lifestyle may be the primary cause. Additionally, differences in one’s own health, the economy, and other underlying factors may have an impact. Therefore, public health interventional measures should be tailored to the needs of different populations.

From 2011 to 2019, the concentration of urine BPA in China was greater than in the United States and Canada, according to the current study. Based on a great number of animal trials and epidemiological studies in Europe and the United States established the daily intake of BPA at 50 g/kg bw/day, but China has no equivalent legislation (66, 67). Furthermore, Wu et al. examined BPA exposure concentrations in natural surface water (freshwater, estuaries, and beaches) in 55 nations. They discovered that BPA exposure concentrations in China were greater than in the United States and Canada (68). The widespread use of BPA in China is the primary reason BPA exposure in the human body is higher than in the United States and Canada, highlighting the need for lowering BPA requirements and use. We collected data from May 2003 and May 2006 as part of the German Environmental Survey (GerES IV). A total of 599 youngsters were chosen, and the BPA level in their urine was 2.66 ng/mL (69). Between 2014 and 2017, the urine levels of BPA in German children were 1.905 ng/mL (70), which was still higher than in Chinese children.

Previous research has demonstrated that BPA has become ubiquitous in the environment as a result of its widespread manufacture, ingestion, and application (71). In addition, BPA environmental sources can be classified as pre-consumer sources for BPA synthesis, BPA-containing items, and post-consumer sources (6). Post-consumer sources, such as garbage disposal, incineration, and sewage discharge, are significant exposure pathways for the general population. BPA levels in landfills in the United States and Japan have been deemed high (72). This study found a link between garbage cleansing and incineration, sewage outflow, and human BPA exposure. As a result, more specific BPA control strategies should be maintained as a public health priority.

Nevertheless, the main limitation of the study is the lack of original work such as experimental or epidemiological investigations. The data of this study were mainly derived from the published literature, which itself resulted from outcome bias. Some provinces have less study data (such as Hongkong, Liaoning) thus the data from a few provinces may not be representative enough of the whole nation. As a result, a population-based epidemiologic survey should be carried out in China to determine the level of BPA exposure there as well as to look into any potential influencing factors, such as lifestyle choices, residential location, health status, age, and gender. We may have omissions in the literature search screening process, resulting in biased results. For example, the study of neonatal urinary BPA exposure by Wang et al. (73). Therefore, we further screened the literature and combined it with epidemiologic studies in the next study. In addition, detection bias may exist in this study. For example, it is detected primarily by ultra-performance liquid chromatography tandem mass spectrometry (UPLC–MS/MS) (32%) and high-performance liquid chromatography (HPLC) with tandem mass spectrometric (MS/MS) (47%), whereas serum BPA is detected by linked immunosorbent assay (ELISA) (32%) and HPLC–MS/MS (46%). The LOD values of all those methods reported from 2004 to 2019 for BPA in human urine samples varied greatly (0.001–1.0 ng/mL). LC-MS/MS is the most sensitive and extensively used method for BPA detection in human urine (74). In addition, it was shown that there was A strong correlation between HPLC/FLD and LC/MS/MS for the determination of BPA levels (75). Even so, the detection bias hardly affected the results due to the high detection consistency and strict quality control.

## 5 Conclusion

Data mining and analysis based on Monte Carlo simulation showed wide fluctuation in the urine of BPA in the Chinese population; serum BPA showed an apparent decrease during 2004–2011, and a noticeable increase during 2012–2019. In addition, the exposure level of urine BPA in the Chinese population was significantly higher than in the United States and Canada. In space, the detection of BPA was mainly in coastal areas, but the scope was extended to the inland. Males’ urine and serum BPA levels were higher than females and children higher than adults. In addition, household garbage cleaning and sewage discharge may affect human BPA levels. In conclusion, this study recommends strengthening the biological detection of BPA and comprehensive management of garbage and sewage discharge to mitigate BPA exposure.

## Data availability statement

The original contributions presented in the study are included in the article/Supplementary material, further inquiries can be directed to the corresponding author.

## Author contributions

WZ and YL: data collection and curation, manuscript writing, and language services. TW: methodology and validation. XZ and JZ: validation. XJ and LL: validation, writing—review and editing, supervision. All authors contributed to the article and approved the submitted version.
