# Supplementary material for: Distribution and potential risk factors of bisphenol a in serum and urine among Chinese from 2004 to 2019
Source: Front Public Health. 2024 Jan 31;12:1196248. doi: 10.3389/fpubh.2024.1196248 (PMC10878132; doi:10.3389/fpubh.2024.1196248)
Supplement: Supplementary file 1 [file Table_1.docx]

Supplementary Material

**Distribution and potential** **risk factors of** **bisphenol A in serum and urine among Chinese from 2004 to 2019**

**Wenjing Zhang^†^,** **Yanting Li^†^, Tao Wang, Xinglin Zhang, Jianzhong Zhang, Xiaoya Ji and Lin Lu***

*** Correspondence:** Lin Lu
[lulin@qdu.edu.cn](mailto:lulin@qdu.edu.cn)

# 1 Supplementary Figures and Tables

## Supplementary Tables

**Table S1** A summary of all included studies concerning urine BPA

| Reference Title | Detection method | Province | Sampling time | Sample Size | AM (ng/mL) | GM (ng/mL) |
| --- | --- | --- | --- | --- | --- | --- |
| Urinary Bisphenol A Concentrations and Their Implications for Human Exposure in Several Asian Countries | HPLC-MS/MS |  | 2006-2007 | 116 |  | 1.10 |
| 双酚A与不明原因复发性流产的1：2病例-对照研究 | 柱前荧光高效液相色谱法检 | Jiangsu | 2008-2010 | 114 | 0.08 |  |
| 双酚A与复发性流产的相关性 | 高效液相色谱 | Jiangsu | 2008-2009 | 60 | 0.06 |  |
| 尿液/血清中双酚 A 水平与不明原因复发性流产风险关系的比较研究 | LC-MS/MS | Jiangsu | 2008-2011 | 162 | 3.37 |  |
| Higher urinary bisphenol A concentrations are associated with risk of unexplained recurrent abortion: evidence from A case-control study in eastern China | LC-MS/MS | Jiangsu | 2008-2011 | 162 | 1.22 |  |
| 环境内分泌干扰物与肥胖及胰岛素抵抗相关性研究 | LC-MS/MS | Shanghai | 2008-2009 | 3423 | 1.15 |  |
| Bisphenol A levels in blood and urine in a Chinese population and the personal factors affecting the levels | HPLC-MS/MS | Eastern and central China |  | 922 | 10.45 |  |
| Urinary bisphenol A concentrations and adiposity measures at age 7 in a prospective birth cohort | LVI-GC-MS / MS | Jiangsu | 2009-2010 | 363 |  | 1.26 |
|  |  |  |  | 229 |  | 1.17 |
|  |  |  |  | 412 |  | 2.66 |
| Urinary bisphenol A concentration and the risk of central obesity in Chinese adults: A prospective study | LC-MS/MS | Shanghai | 2009-2013 | 764 | 1.23 |  |
| Diabetes genetic risk score modifies effect of bisphenol A exposure on deterioration in glucose metabolism | LC-MS/MS | China | 2009-2013 | 1968 | 0.80 |  |
| Association of Bisphenol A Exposure With Hypertension and Early Macrovascular Diseases in Chinese Adults | LC-MS/MS | Shanghai | 2009 | 3246 | 1.17 |  |
| Bisphenol A exposure in relation to altered lipid profile and dyslipidemia among Chinese adults: A repeated measures study | LC-MS/MS | Shanghai | 2009-2013 | 1326 | 1.52 |  |
|  |  |  | 2009-2013 | 1872 | 1.17 |  |
| 江苏省某县两岁幼儿双酚 Ａ 暴露水平评估及影响因素分析 | 高效 液 相 色谱荧光检测仪 | Jiangsu | 2009-2010 | 365 |  | 0.77 |
| 双酚A职业暴露对健康影响的研究 | HPLC-MS/MS | Jiangsu |  | 21 |  | 0.86 |
| 典型环境内分泌干扰物的人体暴露及健康风险评价 | HPLC-MS/MS | China | 2010 | 109 | 3.83 |  |
| 松花江流域内分泌干扰物的环境暴露水平研究 | LC-MS/MS |  |  | 116 | 3.86 |  |
|  |  | Heilongjiang | 2010 | 64 | 3.76 |  |
|  |  | Shanghai |  | 26 | 4.08 |  |
|  |  | Guangdong |  | 26 | 3.87 |  |
| 2岁幼儿双酚A暴露与体格发育的关联：基于莱州湾出生队列研究 | HPLC-MS/MS | Shandong | 2010-2013 | 219 | 0.50 |  |
| 双酚A职业接触限值的研制 | UPLC –MS-MS |  | 2010 | 109 | 9.28 |  |
| Urinary bisphenol A (BPA)concentrations and exposure predictors among pregnant women in the Laizhou Wan Birth Cohort (LWBC), China | HPLC-MS/MS | Shandong | 2010-2013 | 506 | 1.11 |  |
| Associations between maternal phenolic exposure and cord sex hormones in male newborns | UPLC –MS-MS | Guangdong | 2010-2011 | 77 | 2.81 |  |
|  |  | Guangdong |  | 60 | 0.80 |  |
| Association between bisphenol A exposure and body mass index in Chinese school children: a cross-sectional study | SPE-UPLC –MS-MS | Shanghai | 2011 | 124 | 0.87 |  |
| Blood and Urinary Bisphenol A Concentrations in Children, Adults, and Pregnant Women from China: Partitioning between Blood and Urine and Maternal and Fetal Cord Blood | HPLC-MS/MS | Tianjin | 2010 | 50 | 1.90 |  |
| Bisphenol A in Urine of Chinese Young Adults: Concentrations and Sources of Exposure | ESI-MS / MS |  | 2010 | 109 | 3.32 |  |
| 尿液双酚A浓度及生活习惯与女童性早熟关系的研究 | HPLC-MS/MS | Shanghai | 2011 | 44 | 2.82 |  |
| The effects of bisphenol A (BPA)exposure on fat mass and serum leptin concentrations have no impact on bone mineral densities in non-obese premenopausal women | LC-MS/MS | Shanghai |  | 246 | 2.27 |  |
| Determination of Nine Environmental Phenols in Urine by Ultra-High-Performance Liquid Chromatography–Tandem Mass Spectrometry | UPLC –MS-MS | China |  | 325 | 3.38 |  |
| 体重正常和肥胖儿童青少年尿中双酚 A 的分析 | UPLC –MS-MS | Shanghai | 2011 | 120 | 1.03 |  |
| 双酚A与儿童青少年肥胖的关系研 | UPLC –MS-MS | Shanghai | 2011 | 87 | 2.49 |  |
| 双酚A与儿童青少年发育关系的研究 | 高效液相色谱法 | Shanghai | 2011-2012 | 166 | 3.78 |  |
| Prenatal exposure to bisphenol a and its analogues (bisphenol F and S) and ultrasound parameters of fetal growth | UPLC –MS-MS | Hubei | 2011-2012 | 322 | 1.55 |  |
| Urine bisphenol A and pubertal development in boys | HPLC-MS/MS | Shanghai | 2011 | 671 | 6.47 |  |
| Association between bisphenol a exposure and idiopathic central precocious puberty (ICPP) among school-aged girls in Shanghai, China | HPLC-MS/MS | Shanghai | 2011-2012 | 136 | 2.49 |  |
| 4-Nonylphenol, bisphenol-A and triclosan levels in human urine of children and students in China, and the effects of drinking these bottled materials on the levels | GC -NCI-MS | Guangdong |  | 287 | 3.41 |  |
| Parental phenols exposure and spontaneous abortion in Chinese population residing in the middle and lower reaches of the Yangtze River. | UPLC–MS/MS | Jiangsu |  | 170 |  | 0.63 |
|  |  |  |  | 170 |  | 1.04 |
| 尿双酚A与女性身体组成成分和骨密度的关系 | LC-MS/MS | Shanghai |  | 279 | 1.60 |  |
| 高效液相色谱-串联质谱法测定人尿液中5种酚类内分泌干扰物 | HPLC-MS/MS | Fujian |  | 40 | 1.40 |  |
| Measurement of phenolic environmental estrogens in human urine samples by HPLC–MS/MS and primary discussion the possible linkage with uterine leiomyoma | HPLC–MS/MS | Jiangsu |  | 29 | 8.50 |  |
| 孕妇尿酚类环境 内分泌干扰物浓度检测的尿样收集方法比较 | HPLC-MS/MS | Shanghai | 2012-2014 | 642 |  | 1.3 |
| 邻苯二甲酸酯类化合物和双酚A暴露对学龄儿童生长发育影响的研究 | UPLC–MS/MS |  | 2012 | 968 |  | 1.20 |
|  |  | Jiangsu |  | 430 |  | 0.77 |
|  |  | Shanghai |  | 268 |  | 0.88 |
|  |  | Zhejiang |  | 270 |  | 2.61 |
| Urinary levels of bisphenol A, F and S and markers of oxidative stress among healthy adult men: Variability and association analysis | HPLC-MS/MS | Hubei | 2012-2014 | 520 | 0.91 |  |
| Exposure of environmental Bisphenol A in relation to routine sperm parameters and sperm movement characteristics among fertile men | HPLC-MS/MS | Guizhou | 2012 | 500 | 0.38 |  |
| Urinary Bisphenol A Concentration and Gestational Diabetes Mellitus in Chinese Women | HPLC-MS/MS | Shanghai | 2012-2013 | 620 | 6.59 |  |
| Exposure to bisphenol A among school children in eastern China: A multicenter cross-sectional study | UPLC–MS/MS |  | 2012 | 666 | 2.80 |  |
|  |  | Shanghai |  | 230 | 3.37 |  |
|  |  | Jiangsu |  | 224 | 1.43 |  |
|  |  | Zhejiang |  | 212 | 3.80 |  |
| Maternal urinary bisphenol A levels and infant low birth weight: A nested case–control study of the Health Baby Cohort in China | UPLC–MS/MS | Hubei | 2012-2014 | 339 |  | 2.06 |
| Urinary bisphenol analogues concentrations and biomarkers of oxidative DNA and RNA damage in Chinese school children in East China: A repeated measures study | UPLC–MS/MS | East China | 2012-2014 | 801 |  | 1.65 |
| 孕妇尿中重金属及双酚类化合物水平与胎儿生长的关联研究 | UPLC–MS/MS | Hubei | 2013-2015 | 845 |  | 0.83 |
|  |  |  |  | 845 |  | 0.77 |
|  |  |  |  | 845 |  | 0.76 |
| 上海市学龄儿童双酚A暴露现状与肥胖关系的初步研究 | 高效液相色法 | Shanghai | 2013-2014 | 246 | 2.03 |  |
| 甲状腺乳头状癌和结节性甲状腺肿患者血清及尿液中碘、双酚A水平变化 | HPLC-MS/MS | Shandong | 2013 | 65 | 4.69 |  |
| 输卵管因子不孕患者体外输尿双酚A浓度与卵母细胞回收和胚胎植入结果较差相关 | HPLC-MS/MS | Zhejiang | 2013-2016 | 351 | 0.95 |  |
| Urinary bisphenol A and incidence of metabolic syndrome among Chinese men: a prospective cohort study from 2013 to 2017 | HPLC-MS/MS | Guangdong | 2013-2017 | 1038 | 7.70 |  |
| Associations of Trimester-Specific Exposure to Bisphenols with Size at Birth: A Chinese Prenatal Cohort Study | UPLC–MS/MS | Hubei | 2013-2015 | 845 | 2.92 |  |
| Associations of female exposure to bisphenol A with fecundability: Evidence from a preconception cohort study | HPLC-MS/MS | Shanghai | 2013-2015 | 700 |  | 1.30 |
| Higher urinary BPA concentration and excessive iodine intake are associated with NG and PTC | HPLC-MS/MS) | Shandong | 2013 | 65 | 3.96 |  |
| Phenolic environmental estrogens in urine and blood plasma from women with uterine leiomyoma: Epidemiological survey | HPLC-MS/MS) | Jiangsu | 2013-2014 | 300 | 8.62 |  |
| 尿液样本中酚类环境雌激素的含量测定及与子宫肌瘤疾病发生相关性初探 | HPLC-MS/MS) | Jiangsu |  | 29 | 8.50 |  |
| 南京育龄期妇女典型环境内分泌干扰物暴露特征分析 | 超高效液相色谱仪 | Jiangsu | 2014-2016 | 96 | 3.31 |  |
| 尿液双酚A含量与接收人工受精妇女妊娠率的相关性研究 | 固相萃取 | Zhejiang | 2014-2015 | 69 | 5.75 |  |
| 北京女性尿液中双酚A及氯代双酚A的浓度和风险评价 | UPLC-MS-MS | Beijing | 2014-2015 | 40 | 1.30 |  |
| 尿液中三氯生、双酚A浓度与不孕妇女临床结局相关性的分析 | HPLC-MS/MS) | Zhejiang | 2014-2015 | 254 | 10.1 |  |
| 孕期尿环境酚类化合物水平与新生儿出生结局的关联研究 | UPLC–MS/MS | Hubei | 2014 | 985 | 9.98 |  |
| 甲状腺肿患者尿液及血清中碘、ＢＰＡ 检测意义研究 | 高效液相串联质谱 | Zhejiang | 2014-2015 | 70 | 4.69 |  |
| Prenatal exposure to bisphenol A and its alternatives and child neurodevelopment at 2 years | UPLC–MS/MS | Hubei | 2014-2015 | 456 | 1.38 |  |
| Trimester-specific, gender-specific, and low-dose effects associated with non-monotonic relationships of bisphenol A on estrone, 17β-estradiol and estriol | UPLC–MS/MS | Hubei | 2014-2015 | 851 | 5.71 |  |
| Exposure assessment of bisphenols in Chinese women during pregnancy: a longitudinal study | liquid–liquid extractions | Hubei | 2014-2015 | 941 | 0.88 |  |
| Bisphenol A and bisphenol S exposures during pregnancy and gestational age e A longitudinal study in China | UPLC-MS/MS | Hubei | 2014-2015 | 850 | 2.48 |  |
| Relationship between bisphenol A exposure and attention-deficit/ hyperactivity disorder: A case-control study for primary school children in Guangzhou, China | HPLC-MS/MS) | Guangdong | 2014-2017 | 250 |  | 1.70 |
| Occurrence and Profiles of the Artificial Endocrine Disruptor Bisphenol A and Natural Endocrine Disruptor Phytoestrogens in Urine from Children in China | LC-MS/MS | Tianjin | 2014 | 256 | 2.47 |  |
| 人体尿液中几种新兴污染物的检测及其与女性多囊卵巢综合征关联研究 | HPLC-MS/MS | Shandong | 2014 | 212 | 1.51 |  |
|  |  |  |  | 107 | 1.75 |  |
| Exposure of children to BPA through dust and the association of urinary BPA and triclosan with oxidative stress in Guangzhou, China | LC-MS/MS | Guangdong | 2014 | 100 |  | 1.08 |
| 学龄儿童双酚 A 暴露现状及其与肥胖关系的研究 | HPLC-MS/MS | Shanghai | 2013-2014 | 250 | 4.64 |  |
| 中国自来水厂进出水和人体尿液中内分泌干扰物的暴露水平研究和风险评价 | UPLC-MS/MS | Heilongjiang | 2015-2017 | 100 | 1.80 |  |
|  |  | Anhui |  | 50 | 0.56 |  |
|  |  | Henan |  | 50 | 1.23 |  |
|  |  | Jiangsu |  | 100 | 2.27 |  |
|  |  | Liaoning |  | 50 | 1.74 |  |
|  |  | Henan |  | 50 | 1.20 |  |
|  |  | Zhejiang |  | 50 | 1.62 |  |
|  |  | Hunan |  | 100 | 2.08 |  |
|  |  | Shanghai |  | 25 | 0.86 |  |
|  |  | Jiangsu |  | 50 | 4.84 |  |
|  |  | Zhejiang |  | 100 | 1.71 |  |
|  |  | [Inner](javascript:;) [Mongolia](javascript:;) |  | 50 | 0.71 |  |
|  |  | Fujian |  | 50 | 3.08 |  |
|  |  | Shaanxi |  | 100 | 0.83 |  |
|  |  | Shandong |  | 50 | 0.74 |  |
|  |  | Heilongjiang |  | 100 | 1.15 |  |
| 双酚A与泌乳素瘤发生的相关性及机制探讨 | 液相色谱仪 | Shandong | 2015 | 60 | 3.71 |  |
| 中国南方大学生对几种典型环境内分泌干扰物的暴露及健康风险评价 | HPLC-MS/MS | Guangdong | 2015-2016 | 169 | 0.71 |  |
| 不同孕期尿双酚 A与孕妇甲状腺功能指标关联的初步研究 | SPE-UPLC-MS/MS | Shanghai | 2015 | 210 | 0.63 |  |
| Association of bisphenol A or bisphenol S exposure with oxidative stress and immune disturbance among unexplained recurrent spontaneous abortion women | UPLC-MS/MS | Shandong | 2015-2016 | 111 | 1.57 |  |
| Urinary bisphenol analogues and triclosan in children from south China and implications for human exposure | HPLC-MS/MS | Guangdong | 2015 | 213 | 0.31 |  |
|  |  | Guangdong |  | 70 | 2.28 |  |
| The association of bisphenol A exposure with premature ovarian insufficiency: a case–control study | UPLC-MS/MS | Zhejiang | 2015-2018 | 186 | 1.343 |  |
| 超高效液相色谱串联质谱检测尿液中6种环境内分泌干扰物方法的建立与应用 | UPLC-MS/MS | Jiangsu |  | 120 | 2.07 |  |
| A study on phthalate metabolites, bisphenol A and nonylphenol in the urine of Chinese women with unexplained recurrent spontaneous abortion | UPLC-MS/MS | Jiangsu |  | 30 | 4.43 |  |
| 双酚 A 暴露与结直肠癌病理特征及肿瘤相关巨噬细胞的相关研究 | LC-MS/MS | Anhui | 2016-2017 | 344 | 2.41 |  |
| 基于UPLC-MS脂质组学分析结直肠癌差异表达脂质分子与双酚A暴露的相关性 | UPLC-MS | Anhui | 2016-2019 | 130 | 1.67 |  |
| 南通市学龄儿童双酚A暴露现状及其与肥胖的关系 | HPLC-MS/MS | Jiangsu | 2016-2017 | 294 | 1.25 |  |
| 双酚A暴露与学龄女童代谢综合征发生的相关性研究 | HPLC-MS/MS | Jiangsu | 2016-2017 | 314 | 2.46 |  |
| 双酚A暴露水平与学龄期女童特发性性早熟风险之间的关系 | HPLC-MS/MS | Jiangsu | 2016-2018 | 65 | 2.53 |  |
| 双酚A暴露与子宫肌瘤关联性初探 | HPLC-MS/MS | Jiangsu | 2016 | 43 | 2.23 |  |
| Association of bisphenol A and its alternatives bisphenol S and F exposure with hypertension and blood pressure: A cross-sectional study in China | HPLC-MS/MS | Hubei | 2016-2018 | 1004 | 0.51 |  |
|  | UPLC-MS/MS | Hubei | 2016-2018 | 615 | 1.82 |  |
| Urinary levels, composition profile and cumulative risk of bisphenols in preschool-aged children from Nanjing suburb, China | HPLC-MS/MS | Jiangsu | 2016 | 80 | 0.61 |  |
| Organophosphate flame retardants and bisphenol A in children's urine in Hong Kong: has the burden been underestimated? | HPLC-MS/MS | Hongkong | 2016 | 31 | 1.97 |  |
| Urinary phthalate metabolites and environmental phenols in university students in South China | ESI-MS/MS | Guangdong | 2016 | 169 | 0.71 |  |
| 液质联用测定 BPs 和神经递质方法的建立及应用研究 | UPLC-MS/MS | Jiangsu | 2016 | 49 | 4.75 |  |
| 尿液双酚A浓度及生活习惯与女童性早熟关系的研究 | HPLC-MS/MS | Hunan | 2017-2018 | 30 | 3.07 |  |
| 孕妇尿中双酚A暴露水平与孕周的关联性分析 | GC-MS / MS | Shanghai | 2017 | 245 |  | 1.21 |
| 双酚A和遗传相互作用与桥本甲状腺炎及甲状腺结节的关联研究 | HPLC-MS/MS | Jiangsu | 2017-2018 | 991 | 1.69 |  |
|  |  |  |  | 711 | 1.99 |  |
| 双酚A与女童ICPP的关系及遗传学机制探讨 | GC-MS / MS | Shanghai | 2017-2018 | 56 | 3.16 |  |
| 厦门市某区253名6～8岁儿童双酚A暴露水平及影响因素分析 | 三重串联四级杆液质联用法 | Fujian | 2017 | 253 | 2.92 |  |
| Associations of urinary phenolic environmental estrogens exposure with blood glucose levels and gestational diabetes mellitus in Chinese pregnant women | UPLC-MS/MS | Tianjin | 2017-2018 | 390 | 0.95 |  |
| Bisphenol A exposure and risk of thyroid nodules in Chinese women: A casecontrol study | HPLC-MS/MS | Jiangsu | 2017-2018 | 711 | 1.67 |  |
| Co-exposure and health risks of parabens, bisphenols, triclosan, phthalate metabolites and hydroxyl polycyclic aromatic hydrocarbons based on simultaneous detection in urine samples from guangzhou, south China | HPLC-MS/MS | Guangdong | 2018 | 240 | 12.9 |  |
|  |  | Guangdong |  | 240 | 8.59 |  |
| Urinary concentration of personal care products and polycystic ovary syndrome: A case-control study | GC-MS/MS | Shandong 、Zhejiang and Shanghai | 2019-2020 | 83 | 1.37 |  |
| 高通量固相萃取-超高效液相色谱-串联质谱法测定人尿中8种环境酚类内分泌干扰物 | UPLC-MS/MS | Beijing | 2019-2020 | 64 | 1.00 |  |
| Maternal and childhood urinary phenol concentrations, neonatal thyroid function, and behavioral problems at 10 years of age: The SMBCS study | GC-MS/MS | Jiangsu | 2019 | 387 | 1.72 |  |
| 柱前荧光衍生-高效液相色谱法测定尿和血清中的环境雌激素 | 高效液相色谱 |  |  | 20 | 1.25 |  |
| Predictors and Variability of Repeat Measurements of Urinary Phenols and Parabens in a Cohort of Shanghai Women and Men | HPLC-MS/MS | Shanghai | 2006 | 50 |  | 0.794 |
|  |  |  |  | 50 |  | 0.888 |

**Table S2** A summary of all included studies concerning serum BPA

| Reference Title | Detection method | Province | Sampling time | Sample Size | AM (ng/mL) | GM (ng/mL) |
| --- | --- | --- | --- | --- | --- | --- |
| 血清双酚A水平的检测及其与性激素水平的关系研究 | 高效液相色谱法 |  |  | 120 | 3.03 |  |
| 柱前荧光衍生-高效液相色谱法测定尿和血清中的环境雌激素 | 高效液相色谱法 |  |  | 10 | 0.11 |  |
| 性早熟女童血清中双酚 Ａ、辛基酚、4-壬基酚测定和分析 | 高效液相色谱法 | Shanghai | 2005 | 100 | 2.21 |  |
| Association between levels of serum bisphenol A, a potentially harmful chemical in plastic containers, and carotid artery intima-media thickness in adolescents and young adults | ELISA | [Taiwan](javascript:;), [China](javascript:;) | 2006-2008 | 886 | 1.72 |  |
| 血清双酚 A 与糖尿病肾病、高血压肾病的关系研究 | ELISA | Chongqing | 2008 | 230 | 2.87 |  |
| 孕期母体双酚A暴露与新生儿出生指标关联的队列研究 | 立固相萃取-同位素内标-高效液相色谱串联质谱 | Anhui | 2008-2010 | 1335 | 0.39 |  |
| 孕妇血清双酚 A水平与甲状腺功能的关联研究 | 固相萃取－高效液相色谱串联质谱法 | Anhui | 2008-2010 | 2414 | 0.72 |  |
| 尿液/血清中双酚 A水平与不明原因复发性流产风险关系的比较研究 | 高效液相色谱 | Jiangsu | 2008-2011 | 108 | 9.12 |  |
| Serum bisphenol A and progression of type 2 diabetic nephropathy: a 6-year prospective study | ELISA | Chongqing | 2008-2014 | 121 | 1.69 |  |
| Serum bisphenol A as a predictor of chronic kidney disease progression in primary hypertension:  a 6-year prospective study | ELISA | Chongqing | 2008 | 302 | 3.41 |  |
| Bisphenol A is not associated with a 5‑year incidence of type 2 diabetes: a prospective nested case–control study | ELISA | Chongqing | 2008-2013 | 232 | 2.29 |  |
| Serum Bisphenol A Concentration and Premature Thelarche in Female Infants Aged 4-month to 2-year | HPLC-MS/MS | Zhejiang | 2009-2011 | 33 |  | 1.7 |
| Associations of cadmium, bisphenol A and polychlorinated biphenyl co-exposure in utero with placental  gene expression and neonatal outcomes | LC-MS/MS | Guangdong | 2009-2011 | 47 | 4.44 |  |
| Blood and Urinary Bisphenol A Concentrations in Children, Adults, and Pregnant Women from China: Partitioning between Blood and Urine and Maternal and Fetal Cord Blood | HPLC-MS | Tianjin | 2010 | 30 | 3.58 |  |
|  |  | Tianjin |  | 10 | 0.47 |  |
|  |  | Jiangxi |  | 10 | 3.18 |  |
|  |  | Tianjin |  | 50 | 0.20 |  |
|  |  | Jiangxi |  | 30 | 0.13 |  |
| 血清双酚A水平与多囊卵巢综合征相关性的初步研究 | ELISA | Hubei | 2011-2012 | 37 | 1.32 |  |
| 孕早期双酚A暴露与学龄前儿童睡眠问题的关联研究 | 固相萃取-同位素内标-高效液相色谱串联质谱法 | Anhui | 2011-2014 | 1259 | 0.46 |  |
| 双酚A在高尿酸血症中的作用及机制研究 | ELISA | Chongqing | 2012 | 482 | 1.48 |  |
| 扩张型心肌病的分子遗传学机制及环境因素研究 | ELISA | Jiangxi | 2012-2013 | 88 | 3.8 |  |
| 孕期母体双酚A暴露及妊娠相关焦虑致学龄前儿童执行功能损伤的队列研究 | 固相萃取-同位素内标-高效液相色谱串联质谱 | Anhui | 2012-2014 | 1681 | 0.56 |  |
| 孕期双酚A暴露与学龄前儿童饮食行为问题关联的出生队列研究 | 固相萃取-同位素内标-高效液相色谱串联质谱法 | Anhui | 2012-2014 | 1743 | 0.75 |  |
| 0-2岁女童乳房早发育与内分泌干扰物双酚A、邻苯二甲酸二乙酯的相关性 | 高效液相色谱法 | Jiangxi | 2012 |  | 20.51 |  |
| 甲状腺乳头状癌和结节性甲状腺肿患者血清及尿液中碘、双酚A水平变化 | HPLC-MS/MS | Shandong | 2013 | 65 | 8.31 |  |
| 双酚 A 与女童单纯乳房早发育及中枢性性早熟关系的临床观察研究 | 高效液相色谱法 | Jiangsu | 2013 | 32 | 8.036 |  |
|  |  | Jiangsu |  | 53 | 9.912 |  |
| 内分泌干扰物双酚Ａ和邻苯二甲酸二乙酯与婴幼儿女童乳房早发育的相关性 | 高效液相色谱法 | Chongqing | 2013-2014 | 60 | 21.8 |  |
| Higher urinary BPA concentration and excessive iodine intake are associated with NG and PTC | HPLC-MS/MS | Shandong | 2013 | 63 | 7.78 |  |
| Maternal serum bisphenol A levels and risk of pre-eclampsia: a nested case–control study | LC-MS/MS | Shanghai | 2013-2014 | 99 | 1.18 |  |
| 环境雌激素双酚 A 与多囊卵巢综合征的相关性研究 | ELISA | Liaoning |  | 103 | 1.866 |  |
| 不育门诊男性精液质量和性功能与双酚A关系的研究 | 高效液相质谱仪 | Shanxi | 2014-2015 | 353 | 6.83 |  |
| 双酚A与不明原因复发性流产发生关系的病例对照研究 | 固相萃取-同位素内标-高效液相色谱串联质谱法 | Chongqing | 2014-2016 | 80 | 5.58 |  |
| 浦东新区224名学龄儿童血清中双酚A水平调查 | 酶水解-高效液相色谱法 | Shanghai | 2014-2015 | 224 | 2.08 |  |
| 环境雌激素双酚 A 对女性生殖功能的影响 | ELISA |  | 2014-2015 | 48 | 8.17 |  |
| 甲状腺肿患者尿液及血清中碘、BPA 检测意义研究 | 高效液相串联质谱 | Zhejiang | 2014-2015 | 70 | 8.12 |  |
| 南昌区近郊区超重与肥胖儿童血清双酚A水平调查 | ELISA | Jiangxi | 2014 | 148 | 2.09 |  |
|  |  |  |  |  | 1.49 |  |
| The correlation between UDP glucuronosyltransferase polymorphisms and environmental endocrine disruptors levels in polycystic ovary syndrome patients | LC-MS/MS | Sichuan | 2014-2016 | 229 |  | 4.76 |
| 免疫亲和柱净化高效液相色谱荧光检测儿童血清中双酚A | 免疫亲和柱净化高效液相色谱荧光 | Jiangxi |  | 56 | 7.39 |  |
| 双酚A与泌乳素瘤发生的相关性及机制探讨 | LC-MS/MS | Shandong | 2015 | 60 | 4.30 |  |
| 环境内分泌干扰物与婴幼儿单纯乳房早发育的临床及转归的相关性研究 | 高效液相色谱法 | Jiangxi | 2015-2016 | 30 | 28.71 |  |
| Association of prenatal exposure to bisphenols and birth size in Zhuang ethnic newborns | UPLC-MS | Guangxi | 2015-2018 | 2023 |  | 2.03 |
| Occurrence and Partitioning of Bisphenol Analogues in Adults’ Blood from China | LC-MS/MS | Zhejiang | 2016 | 19 | 0.67 |  |
| 银杏叶片联合阿托伐他汀对冠心病伴代谢综合征患者血清BPA、ADMA、visfatin水平及TLR4/NF-κB信号通路的影响 | ELISA | Liaoning | 2018-2020 | 50 | 0.68 |  |
| Associations of serum bisphenol A levels with incident chronic kidney disease risk | HPLC-MS | Zhejiang | 2018 | 1124 | 3.42 |  |

**Table S3** Urine and serum BPA concentrations (ng/mL) in Chinese population

| Variable | Years | GM | SEM | N | N_ref_ |
| --- | --- | --- | --- | --- | --- |
| Urine BPA | 2006-2008 | 1.70 | 0.03 | 4959 | 7 |
|  | 2009-2011 | 1.30 | 0.01 | 14278 | 25 |
|  | 2012-2014 | 1.84 | 0.01 | 19624 | 38 |
|  | 2015-2017 | 1.32 | 0.02 | 8769 | 27 |
|  | 2018-2019 | 2.90 | 0.06 | 1014 | 4 |
| Serum BPA | 2004-2007 | 1.78 | 0.04 | 1166 | 4 |
|  | 2008-2011 | 1.07 | 0.02 | 6248 | 12 |
|  | 2012-2015 | 1.66 | 0.02 | 7642 | 21 |
|  | 2016-2019 | 2.54 | 0.04 | 1193 | 3 |

GM: geometric mean; SEM: standard error of mean; N: Sample Size; N_ref_: number of references

**Table S4** Data in geometrical mean of urine BPA (ng/mL) by provinces

| Years | Provinces | GM | N | N_ref_ |
| --- | --- | --- | --- | --- |
| 2008-2011 | Jiangsu | 1.35 | 1888 | 7 |
|  | Shanghai | 1.27 | 12251 | 13 |
|  | Heilongjiang | 2.75 | 64 | 1 |
|  | Guangdong | 1.75 | 163 | 3 |
|  | Shandong | 1.05 | 725 | 2 |
|  | Tianjin | 1.65 | 50 | 1 |
|  | Hubei | 1.38 | 322 | 1 |
| 2012-2019 | Jiangsu | 1.66 | 4666 | 15 |
|  | Shanghai | 1.69 | 3769 | 12 |
|  | Heilongjiang | 1.40 | 200 | 1 |
|  | Guangdong | 3.50 | 2776 | 8 |
|  | Shandong | 1.80 | 670 | 6 |
|  | Tianjin | 1.39 | 646 | 2 |
|  | Hubei | 1.46 | 9941 | 11 |
|  | Fujian | 2.15 | 343 | 3 |
|  | Zhejiang | 2.57 | 1562 | 8 |
|  | Guizhou | 1.24 | 500 | 1 |
|  | Beijing | 1.06 | 104 | 2 |
|  | Anhui | 1.80 | 524 | 3 |
|  | Henan | 1.21 | 50 | 1 |
|  | Liaoning | 1.55 | 50 | 1 |
|  | Hunan | 1.68 | 180 | 2 |
|  | [Inner](javascript:;) [Mongolia](javascript:;) | 0.85 | 50 | 1 |
|  | Shaanxi | 0.87 | 100 | 1 |
|  | Hongkong | 1.71 | 31 | 1 |

GM: geometric mean; N: Sample Size; N_ref_:Number of references

**Table S5**  Data in geometrical mean of serum BPA (ng/mL) by provinces

| Years | Provinces | GM | N | N_ref_ |
| --- | --- | --- | --- | --- |
| 2006-2011 | Taiwan, China | 1.73 | 886 | 1 |
|  | Chongqing | 2.24 | 885 | 4 |
|  | Anhui | 0.89 | 5088 | 3 |
|  | Jangsu | 5.03 | 108 | 1 |
|  | Shanghai | 1.91 | 100 | 1 |
|  | Zhejiang | 5.27 | 33 | 1 |
|  | Guangdong | 3.18 | 44 | 1 |
|  | Tianjin | 0.92 | 90 | 1 |
|  | Jiangxi | 0.77 | 40 | 1 |
|  | Hubei | 1.21 | 37 | 1 |
| 2012-2019 | Chongqing | 1.89 | 622 | 3 |
|  | Anhui | 0.91 | 3424 | 2 |
|  | Jiangsu | 9.14 | 85 | 1 |
|  | Shanghai | 1.61 | 323 | 2 |
|  | Zhejiang | 2.86 | 1213 | 3 |
|  | Jiangxi | 5.46 | 174 | 3 |
|  | Shandong | 5.80 | 188 | 3 |
|  | Liaoning | 1.33 | 153 | 2 |
|  | Sichuan | 4.75 | 229 | 1 |
|  | Shanxi | 5.28 | 353 | 1 |
|  | Guangxi | 2.19 | 2023 | 1 |

GM: geometric mean; N: Sample Size; N_ref_: Number of references

**Table S6** Data in geometrical mean of urine and serum BPA (ng/mL) by gender

| Variable | Survey time | Males | | | | Females | | | | *P* |
| --- | --- | --- | --- | --- | --- | --- | --- | --- | --- | --- |
|  |  | GM | SEM | N | N_ref_ | GM | SEM | N | N_ref_ |  |
| Urine BPA | Total | 2.12 | 0.02 | 7377 | 22 | 1.77 | 0.01 | 19623 | 53 | <0.001 |
|  | 2006-2008 | 1.28 | 0.03 | 1997 | 3 | 1.02 | 0.02 | 3258 | 7 | <0.001 |
|  | 2009-2011 | 1.87 | 0.06 | 1293 | 6 | 1.30 | 0.03 | 2002 | 10 | <0.001 |
|  | 2012-2014 | 2.06 | 0.03 | 3568 | 11 | 1.66 | 0.02 | 11772 | 27 | <0.001 |
|  | 2016-2018 | 1.20 | 0.07 | 439 | 2 | 1.42 | 0.03 | 2204 | 8 | <0.001 |
|  | 2019 | - | - | - | - | 1.48 | 0.05 | 387 | 1 | - |
| Serum BPA | Total | 3.03 | 0.06 | 919 | 7 | 1.07 | 0.02 | 10143 | 21 | <0.001 |
|  | Before2010 | 1.56 | 0.07 | 377 | 2 | 1.11 | 0.02 | 4603 | 7 | <0.001 |
|  | 2011-2012 | 3.29 | 0.14 | 55 | 1 | 0.86 | 0.02 | 4743 | 5 | <0.001 |
|  | 2013-2014 | 5.24 | 0.05 | 477 | 3 | 3.94 | 0.07 | 787 | 8 | <0.001 |
|  | 2015-2016 | 0.62 | 0.34 | 10 | 1 | 0.75 | 0.34 | 10 | 1 | <0.001 |

GM: geometric mean; SEM: standard error of mean; N: Sample Size; N_ref_: number of references

**Table S7** Data in geometrical mean of urine and serum (ng/mL) by age

| Variable | Age | GM | SEM | N | N_ref_ | *P* |
| --- | --- | --- | --- | --- | --- | --- |
| Urine BPA | Adult (≤18) | 1.50 | 0.01 | 35972 | 63 | <0.001 |
|  | Children (＞19) | 1.80 | 0.02 | 8285 | 31 |  |
|  | 0-6 | 1.12 | 0.04 | 1240 | 9 | <0.001 |
|  | 7-18 | 2.12 | 0.02 | 7045 | 22 |  |
|  | 19-45 | 1.85 | 0.01 | 17188 | 44 |  |
|  | 45－ | 0.85 | 0.02 | 3768 | 5 |  |
| Serum BPA | Children (≤18) | 2.88 | 0.07 | 955 | 11 | <0.001 |
|  | Adult (>19) | 1.36 | 0.01 | 14936 | 27 |  |
|  | 0-6 | 6.44 | 0.21 | 227 | 6 | <0.001 |
|  | 7-18 | 2.28 | 0.06 | 700 | 4 |  |
|  | 19-30 | 0.98 | 0.04 | 1963 | 5 |  |
|  | 31-40 | 1.25 | 0.08 | 803 | 3 |  |
|  | 41-50 | 2.51 | 0.46 | 47 | 2 |  |
|  | 51－ | 2.25 | 0.06 | 553 | 3 |  |

GM: geometric mean; SEM: standard error of mean; N: Sample Size; N_ref_: number of references

**Table S8**. The association between urine BPA concentration in Chinese and the external environment

| Years | provience | Urine BPA level（ng/mL) | Volume of garbage disposal (The million tons) | Domestic sewage (one hundred thousand tons) | Waste incineration content (Thousand tons/day) |
| --- | --- | --- | --- | --- | --- |
| 2008 | Jiangsu | 1.19 | 0.9345 | - | - |
|  | Shanghai | 1.1 | 0.676 | - | - |
| 2009 | Jiangsu | 1.41 | 0.9573 | 5.704 | 1.4068 |
|  | Shanghai | 1.16 | 0.71 | 2.144 | 0.2575 |
| 2010 | Shanghai | 2.74 | 0.732 | 1.982 | 0.2575 |
|  | Heilongjiang | 2.75 | 0.7824 | 3.32 | 0.05 |
|  | Guangdong | 1.75 | 1.9386 | 6.24 | 1.1743 |
|  | Shandong | 1.05 | 0.992 | 3.254 | 0.82 |
|  | Tianjin | 1.65 | 0.1837 | 1.098 | 0.18 |
| 2011 | Shanghai | 2.55 | 0.704 | 1.81866 | 0.15 |
|  | Hubei | 1.38 | 0.7363 | 4.61323 | 0.4409 |
| 2012 | Jiangsu | 1.2 | 1.2101 | 5.72502 | 2.1269 |
|  | Shanghai | 1.96 | 0.716 | 1.77504 | 0.25 |
|  | Guangdong | 2.81 | 2.1369 | 9.50426 | 1.5835 |
|  | Hubei | 1.36 | 0.7166 | 4.61247 | 0.8012 |
|  | Fujian | 2.41 | 0.4938 | 3.51263 | 0.955 |
|  | Zhejiang | 2.66 | 1.055 | 3.89175 | 2.2365 |
|  | Guizhou | 1.25 | 0.2357 | 1.99737 | 0 |
| 2013 | Jiangsu | 4.82 | 1.2027 | 5.58698 | 2.347 |
|  | Shanghai | 1.65 | 0.735 | 1.73181 | 0.63 |
|  | Guangdong | 4.66 | 2.0921 | 9.03799 | 2.1345 |
|  | Shandong | 3.18 | 1.0074 | 4.13286 | 1.18 |
|  | Hubei | 1.07 | 0.7458 | 4.55112 | 1 |
|  | Zhejiang | 1.08 | 1.1233 | 3.76253 | 2.6803 |
| 2014 | Jiangsu | 2.4 | 1.3534 | 5.27877 | 2.9817 |
|  | Guangdong | 1.94 | 2.2142 | 8.64345 | 2.3235 |
|  | Shandong | 1.55 | 0.9585 | 3.85112 | 1.47 |
|  | Tianjin | 1.95 | 0.2159 | 0.80459 | 0.43 |
|  | Hubei | 2.29 | 0.7393 | 4.46947 | 1.095 |
|  | Zhejiang | 4.72 | 1.2291 | 3.60421 | 2.9705 |
|  | Beijing | 1.21 | 0.7338 | 0.82194 | 0.52 |
| 2015 | Jiangsu | 2.14 | 1.4561 | 4.99624 | 3.2137 |
|  | Shanghai | 0.72 | 0.6132 | 1.41238 | 0.83 |
|  | Heilongjiang | 1.4 | 0.023 | 2.81478 | 0.18 |
|  | Guangdong | 0.85 | 2.3204 | 8.34653 | 2.577 |
|  | Shandong | 1.6 | 1.3775 | 4.20274 | 1.835 |
|  | Fujian | 2.21 | 0.6081 | 3.3236 | 1.23 |
|  | Zhejiang | 1.33 | 1.3326 | 3.47234 | 3.2435 |
|  | Anhui | 0.66 | 0.4919 | 4.27176 | 0.775 |
|  | Henan | 1.21 | 0.8918 | 3.78583 | 0.485 |
|  | Liaoning | 1.55 | 0.9332 | 2.64778 | 0.178 |
|  | Hunan | 1.59 | 0.6382 | 5.31461 | 0.16 |
|  | [Inner](javascript:;) [Mongolia](javascript:;) | 0.85 | 0.3453 | 1.44724 | 0.135 |
|  | Shanxi | 0.87 | 0.5227 | 1.91786 | 0 |
| 2016 | Jiangsu | 1.63 | 1.5623 | 3.611 | 3.3093 |
|  | Guangdong | 1.03 | 2.391 | 5.42042 | 3.0045 |
|  | Hubei | 1 | 0.8801 | 2.72522 | 1.2521 |
|  | Anhui | 1.92 | 0.54 | 2.82284 | 0.905 |
| 2017 | Jiangsu | 1.52 | 1.7347 | 3.78744 | 3.8979 |
|  | Shanghai | 1.36 | 0.7431 | 0.53275 | 1.33 |
|  | Tianjin | 1.11 | 0.3069 | 0.29009 | 0.55 |
|  | Fujian | 1.34 | 0.657 | 2.19814 | 1.51 |
|  | Hunan | 2.28 | 0.7549 | 2.47472 | 0.46 |
| 2018 | Guangdong | 5.62 | 3.0354 | 5.61767 | 5.3872 |
| 2019 | Jiangsu | 1.48 | 1.8096 | 3.54553 | 4.681 |
|  | Beijing | 0.98 | 1.0112 | 0.38233 | 1.709 |

## Supplementary Figures


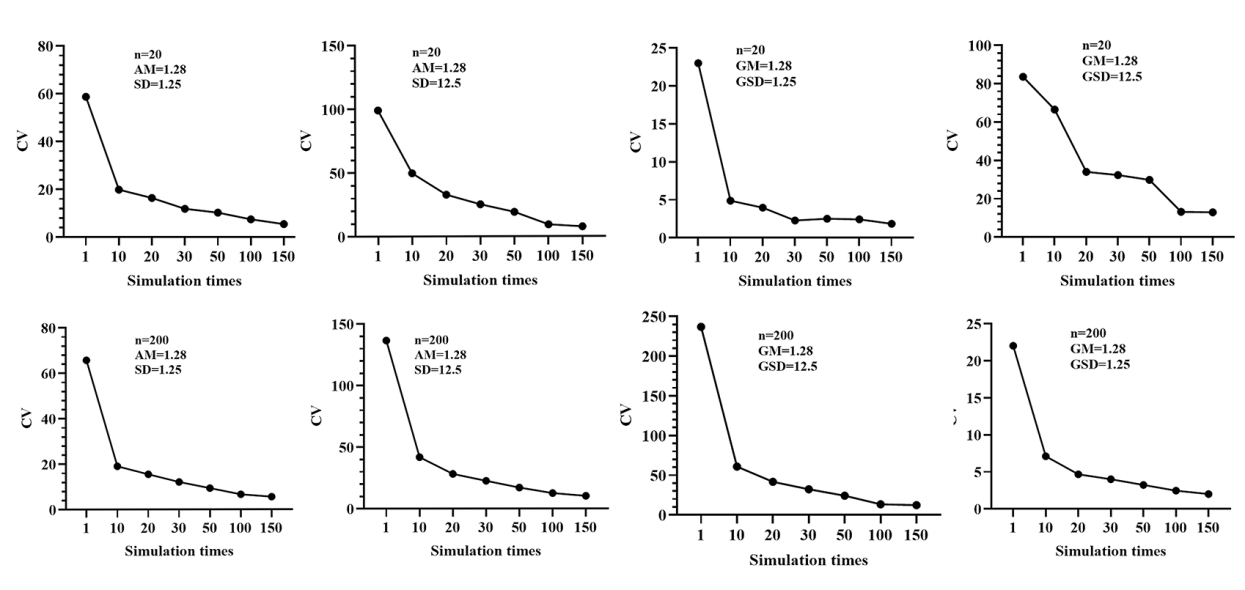


**Supplementary Figure 1.** The variability for different number of simulations of different data. X axis is the number of simulation (1, 10, 20, 30, 100, and 150). Y axis is CV (coefficient variation). CV is proportional to the standard deviation and sample size. The number of simulations is considered reasonably accurate when CV is less than or equal to 15%
